# Specialized digestive mechanism for an insect-bacterium gut symbiosis

**DOI:** 10.1093/ismejo/wrad021

**Published:** 2024-01-10

**Authors:** Junbeom Lee, Bohyun Jeong, Jeongtae Kim, Jae H Cho, Jin H Byeon, Bok L Lee, Jiyeun K Kim

**Affiliations:** Metabolomics Research Center for Functional Materials, Kyungsung University, Busan 48434, South Korea; Department of Microbiology, Kosin University College of Medicine, Busan 49267, South Korea; Department of Anatomy, Kosin University College of Medicine, Busan 49267, South Korea; Host Defense Protein Laboratory, College of Pharmacy, Pusan National University, Busan 46241, South Korea; Host Defense Protein Laboratory, College of Pharmacy, Pusan National University, Busan 46241, South Korea; Host Defense Protein Laboratory, College of Pharmacy, Pusan National University, Busan 46241, South Korea; Department of Microbiology, Kosin University College of Medicine, Busan 49267, South Korea

**Keywords:** Riptortus pedestris, Burkholderia, symbiosis, midgut, fibers

## Abstract

In *Burkholderia*-*Riptortus* symbiosis, the host bean bug *Riptortus pedestris* harbors *Burkholderia* symbionts in its symbiotic organ, M4 midgut, for use as a nutrient source. After occupying M4, excess *Burkholderia* symbionts are moved to the M4B region, wherein they are effectively digested and absorbed. Previous studies have shown that M4B has strong symbiont-specific antibacterial activity, which is not because of the expression of antimicrobial peptides but rather because of the expression of digestive enzymes, mainly cathepsin L protease. However, in this study, inhibition of cathepsin L activity did not reduce the bactericidal activity of M4B, indicating that there is an unknown digestive mechanism that renders specifically potent bactericidal activity against *Burkholderia* symbionts. Transmission electron microscopy revealed that the lumen of symbiotic M4B was filled with a fibrillar matter in contrast to the empty lumen of aposymbiotic M4B. Using chromatographic and electrophoretic analyses, we found that the bactericidal substances in M4B existed as high-molecular-weight (HMW) complexes that were resistant to protease degradation. The bactericidal HMW complexes were visualized on non-denaturing gels using protein- and polysaccharide-staining reagents, thereby indicating that the HMW complexes are composed of proteins and polysaccharides. Strongly stained M4B lumen with Periodic acid–Schiff (PAS) reagent in M4B paraffin sections confirmed HMW complexes with polysaccharide components. Furthermore, M4B smears stained with Periodic acid–Schiff revealed the presence of polysaccharide fibers. Therefore, we propose a key digestive mechanism of M4B: bacteriolytic fibers, polysaccharide fibers associated with digestive enzymes such as cathepsin L, specialized for *Burkholderia* symbionts in *Riptortus* gut symbiosis.

The bean bug (*Riptortus pedestris*), a major pest of soybean in East Asia, harbors an extracellular *Burkholderia* symbiont in a midgut region called M4. *Riptortus* has morphologically distinct midgut regions: M1, M2, M3, M4B, and M4 (Fig. 1A) [1, 2]. When *Burkholderia* cells enter the M4 region during the second instar stage of *Riptortus*, the junction between M3 and M4B is completely closed, and the M4 and M4B regions function as symbiotic organs for *Burkholderia* symbionts [3]. In contrast to M4 bearing numerous crypts (sac-like structures) wherein *Burkholderia* symbionts proliferate, the M4B region is devoid of crypts and possesses potent antibacterial activity against the symbiotic *Burkholderia*, which is induced by symbiotic association [4]. Previous studies have suggested that M4B is a digestive organ for *Burkholderia* symbionts [4, 5]. Excess symbionts after filling the M4 region flow to the M4B region and are digested to provide nutrients to the host, thereby contributing to growth, fitness, immunity, and egg production [6-8].

**Figure 1:**
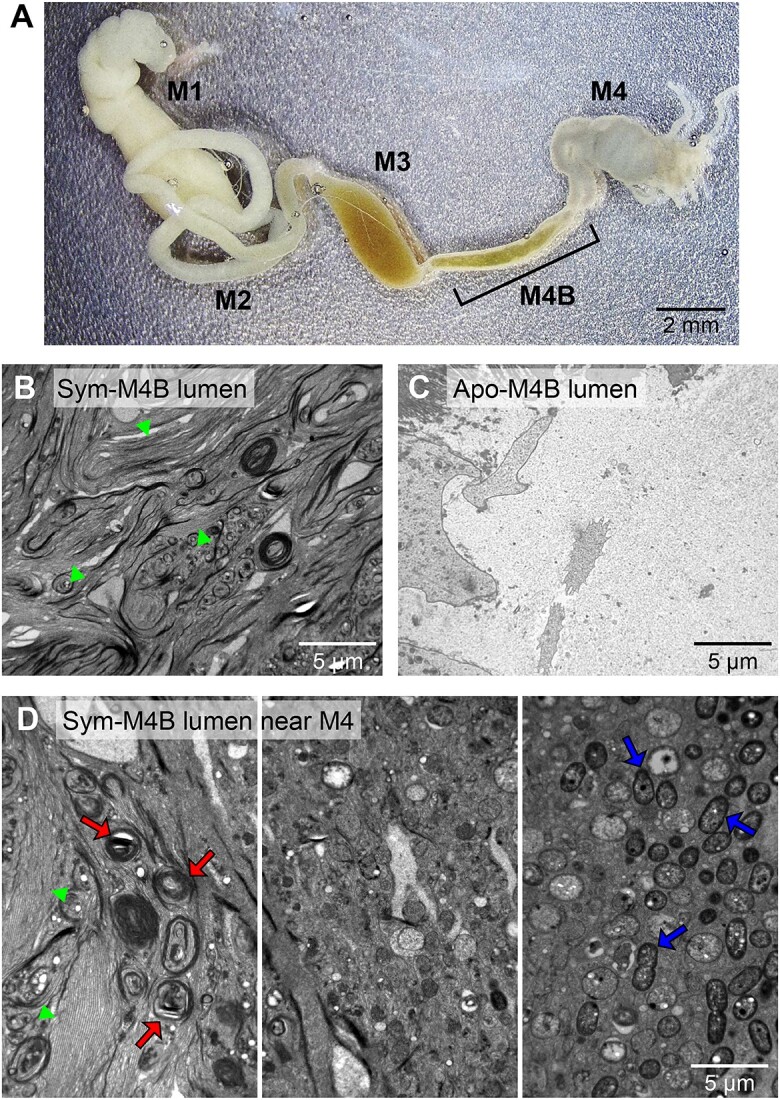
(A) Midgut regions of bean bug. *Burkholderia* cells uptaken by mouth move through midgut regions in the order of M1, M2, M3, M4B, and M4. M4 midgut region is wherein they colonize and proliferate. After filling up the M4, excess *Burkholderia* symbionts flow back to M4B where they are digested and absorbed by the host. (B) TEM image of M4B lumen of symbiotic bean bug. TEM image was obtained from the M4B specimen of a bean bug harboring *Burkholderia* symbiont in M4. The fibrillar matter is indicated with green arrowheads. (C) TEM image of M4B lumen of aposymbiotic bean bug. The M4B specimen was prepared from a bean bug without *Burkholderia* symbionts. (D) TEM images of M4B lumen located close to M4 of symbiotic bean bug. Intact *Burkholderia* cells coming from M4 (blue arrows in the third panel) are lysed as they move toward the fibrillar matter of M4B. Remnants of dissolved *Burkholderia* cells in the fibrillar matters are indicated by red arrows in the first panel. The fibrillar matter is indicated with green arrowheads. Scale bars are shown on the images.

To understand the digestive mechanism of M4B, we initially examined the expression of antimicrobial peptides and found that antimicrobial peptide expression was not upregulated in symbiotic M4B [4]. Comparative transcriptomic analyses of genes expressed in symbiotic and aposymbiotic midgut regions have identified several M4B genes that may play a role in killing and digesting *Burkholderia* symbionts, including cathepsin L proteases, zinc carboxypeptidase, and GPI-anchor transamidase [9]. Byeon *et al*. [10] identified a cathepsin L (25 kDa) as a major protein in the M4B fraction with antibacterial activity and showed that recombinant cathepsin L exerted antibacterial activity against symbiotic *Burkholderia* cells. However, we could not regard the action of cathepsin L as a sole bactericidal and digestive mechanism of M4B. Because when the activity of cathepsin L in the M4B fraction was inhibited by cathepsin L inhibitors, the M4B fraction retained strong bactericidal activity (Fig. S1). Therefore, we continued to investigate the key digestive mechanism of the M4B region, which can specifically respond to potent bactericidal activity against *Burkholderia* symbionts.

When the transmission electron microscopic (TEM) images of the symbiotic organs of *Riptortus* were analyzed, the M4B tissue showed long villi when compared with the M4 tissue, supporting M4B function of absorbing nutrients after digesting symbionts (Fig. S2). The lumen of symbiotic M4 was filled with symbionts with a clear background (Fig. S2A). However, the lumen of the symbiotic M4B was filled with a fibrillar matter (Fig. 1B). In contrast, the lumen of aposymbiotic M4B was clear with few substances, indicating that the fibrillar matter of M4B was specific for symbiosis (Fig. 1C). A series of TEM images of the M4B lumen, close to M4 region, revealed that intact *Burkholderia* symbionts originating from M4 were gradually lysed as they approached the fibrillar matter (Fig. 1D).

To characterize the bactericidal substances of M4B, we performed size-exclusion chromatography. Antibacterial activity against symbionts was detected in the first-peak fractions (Fig. S4), suggesting that bactericidal substances existed as high-molecular-weight (HMW) complexes (Fig. 2A). To dissociate the HMW complex, the eluate fractions with antibacterial activity were treated with protease K and subjected to the second size-exclusion chromatography. Despite protease treatment, the first-peak fractions retained high bactericidal activity, similar to that of the control chromatography (Fig. 2B). These results indicated that the HMW complex of M4B is neither dissociated nor lost its bactericidal activity after protease K treatment.

**Figure 2:**
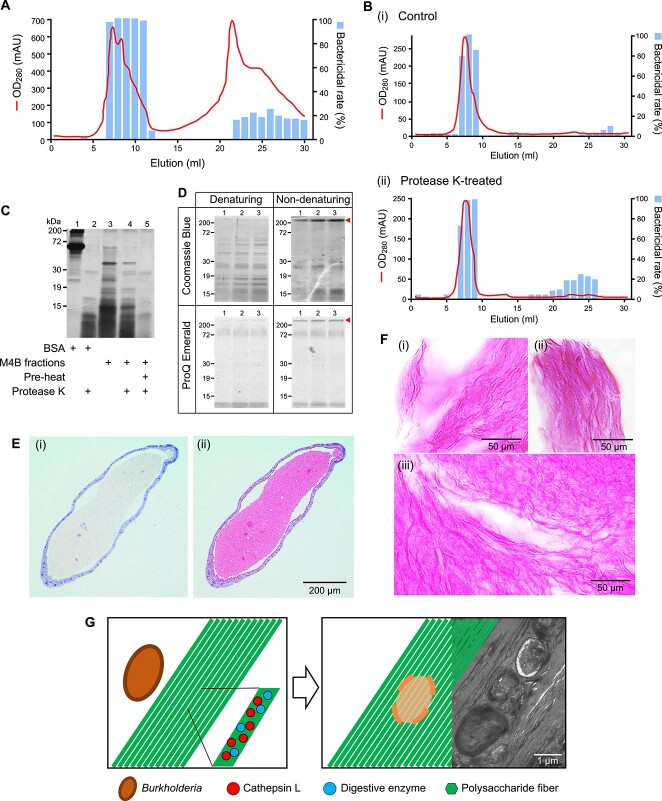
(A) Size-exclusion chromatography of M4B lysate. The M4B lysate prepared from 200 bean bugs was separated by a Superdex 200 (1 × 30 cm) column according to their size and collected 1 mL of eluate per fraction. The protein contents of eluates were detected by UV absorbance at 280 nm (OD_280_, red line). Bactericidal activity of each fraction was determined by optical density at 600 nm of a 12-h culture of *Burkholderia* symbiont after treating with fractions. The bactericidal rate (light blue column) was calculated using the following formula: {1 – (OD_600_ of sample / OD_600_ of control)} × 100. (B) Size-exclusion chromatography of M4B fractions with protease K treatment. Eluates with bactericidal activity from the first size exclusion chromatography (5 mL) were concentrated. Prior to loading onto a Superdex 200 column, half of the concentrates were non-treated for use as a control (i), and the other half was treated with protease K (100 μg/mL) for 1 h (ii). (C) SDS-PAGE analysis of M4B fractions with protease K treatment. BSA was treated with protease K to verify the proteolytic activity (Lanes 1 and 2). Proteins of M4B fractions without treatment (Lane 3), protease K-treatment (Lane 4), and heat-denaturation and protease K treatment (Lane 5) were separated on SDS-PAGE and visualized by Coomassie Brilliant Blue staining. Protein bands of protease K are shown at 29 kDa in Lanes 2, 4, and 5. (D) Denaturing and non-denaturing SDS-PAGE analyses of M4B fractions with protein or polysaccharide staining. Proteins and polysaccharides on the denaturing and non-denaturing SDS-PAGE gels were visualized by Coomassie Brilliant Blue staining and ProQ emerald 300 staining, respectively. The HMW complexes are shown in non-denaturing gels (red arrowheads). (E) H&E and PAS staining of M4B paraffin sections. The paraffin-embedded M4B section was stained with H&E (i) or hematoxylin and PAS (ii). M4B tissues were stained with hematoxylin, showing blue-purple color. However, the M4B lumen was specifically stained with PAS, showing magenta color. (F) PAS staining of M4B smear. The M4B lumen contents were smeared on slide glasses and stained with PAS. Polysaccharide fibers are visualized in magenta color (i–iii). (G) Schematic illustration of the lysis of *Burkholderia* symbiont by bacteriolytic fibers. As coming from M4 to M4B, *Burkholderia* symbionts move toward polysaccharide fibers tightly associated with digestive enzymes, named bacteriolytic fibers. The contact with the bacteriolytic fibers induces lysis and digestion of *Burkholderia* symbionts, and consequently absorption by M4B. Scale bars are shown on the images.

To examine whether the M4B proteins of HMW complex are protected from protease K degradation, SDS-PAGE was performed using the first-peak M4B fractions with bactericidal activity. Treatment with protease K completely degraded the bovine serum albumin (BSA) and heat-denatured M4B fraction (Fig. 2C, Lanes 2 and 5). However, protease K failed to degrade the proteins in the M4B fraction (Fig. 2C, Lane 4), showing protein bands similar to those of the untreated control (Fig. 2C, Lane 3). We speculated that the formation of the HMW complex in M4B protects the proteins from proteolytic activity. To confirm the existence of the complex and identify its structural components, the denatured and non-denatured M4B fractions were analyzed by SDS-PAGE. HMW complexes were visualized only in the non-denaturing gels with Coomassie Brilliant Blue protein staining (Fig. 2D, upper panel) and with ProQ Emerald 300 saccharide staining (Fig. 2D, bottom panel). Because protein bands in the denaturing gels were not stained with ProQ Emerald 300 staining, the HMW complex is most likely composed of proteins and polysaccharides rather than glycoproteins. We also verified that DNA is not a component of the complex (Fig. S3).

Because the fibrillar matter, abundantly observed in the TEM images of symbiotic M4B, may consist of the bactericidal HWM complex, Periodic acid–Schiff (PAS) reagent was used to specifically stain saccharide components in the M4B lumen. When the M4B paraffin sections were stained with hematoxylin and eosin (H&E) and PAS, the contents of the M4B lumen were strongly stained with PAS, when compared with the H&E staining (Fig. 2E). When M4B smears were stained with PAS, polysaccharide fibers were observed (Fig. 2F). These findings imply that the fibrillar matters shown in the TEM images may be further assembled to form fibers made up of polysaccharides and proteins.

We propose a novel digestive mechanism specialized for *Burkholderia* symbionts in *Riptortus* gut symbiosis: bacteriolytic fibers. Bacteriolytic fibers are polysaccharide fibers tightly associated with digestive proteins, such as cathepsin L, zinc carboxypeptidase, and GPI-anchor transamidase. In the M4B midgut, bacteriolytic fibers may provide locally concentrated digestive enzymes, thereby inducing effective lysis of *Burkholderia* symbionts. Although speculative, the physical pressure of bacteriolytic fibers on incoming *Burkholderia* symbionts may also promote cell lysis. The envelope of *Burkholderia* cells undergoes dramatic changes as they become gut symbionts of *Riptortus* [10-15]. These changes may allow *Burkholderia* symbionts to be easily lysed by bacteriolytic fibers. Therefore, *Burkholderia*-*Riptortus* gut symbiosis demonstrates an effective mechanism for nutrient uptake by the host through adaptive changes in *Burkholderia* symbionts in the M4 midgut and symbiont-specialized bacteriolytic fibers in the M4B midgut.

## Conflicts of interest

None declared.

## Funding

A National Research Foundation of Korea (NRF) grant funded by the South Korean government (MSIT) (NRF-2021R1A2C1006793); NRF Basic Science Research Program grant funded by the Ministry of Education (RS-2023-00245952).

## Data availability

All data generated or analyzed during this study are included in this published article and its supplementary information files.

## Supplementary Material

M4B_supplementary_information_wrad021
