# Supplementary material for: Specialized digestive mechanism for an insect-bacterium gut symbiosis
Source: ISME J. 2024 Jan 10;18(1):wrad021. doi: 10.1093/ismejo/wrad021 (PMC10811733; doi:10.1093/ismejo/wrad021)

Supplementary Information

Specialized digestive mechanism for an insect-bacterium gut symbiosis

Junbeom Lee, Bohyun Jeong, Jeongtae Kim, Jae Hyun Cho, Jin Hee Byeon, Bok Luel Lee, Jiyeun Kate Kim

Supplementary Methods

Bean bug rearing. *Riptortus pedestris* was reared in our insect laboratory at 28°C under a long-day regime of 16 h light and 8 h dark. When the newborn nymphs molted to the 2nd instar, *Burkholderia insecticola* inoculating solution was provided with wet cotton balls in a petri dish. *Burkholderia* inoculating solution was prepared using exponential-phase *Burkholderia* cells suspended in distilled water containing 0.05% ascorbic acid at a final concentration of 10^7 cells/mL. Upon reaching adulthood, the insects were transferred to larger containers (35 cm length, 35 cm width, and 40 cm height), wherein soybean plant pots were placed for feeding and cotton pads were attached to the cage walls for egg laying. The eggs were collected daily and transferred to new cages for hatching. The care and treatment of *Burkholderia* cells and insects in all procedures strictly followed the guidelines of the Kosin University Institutional Animal Care and Use Committee (IACUC) and the Living Modified Organ (LMO) Committee.

Transmission electron microscopy. M4B and M4 midgut regions of symbiotic insects and aposymbiotic insects were dissected from 5th instar nymphs (18-day-old) and pre-fixed with 2.5% glutaraldehyde in sodium cacodylate buffer (SCB, 0.1 M sodium cacodylate trihydrate, pH 7.4) at 4°C for 18 h. After pre-fixation, the samples were washed thrice with SCB and post-fixed with 1% osmium tetroxide in SCB for 1 h at room temperature (approximately 23 - 25°C). Then the post-fixation solution was replaced with 0.5% uranyl acetate solution and incubated for overnight at room temperature. After incubation, the samples were dehydrated by immersion in an ethanol-propylene oxide series and embedded in Epon 812 resin. The embedded samples were trimmed and sectioned using an ultramicrotome (Reichert SuperNova, Leica, Germany). The sections were observed under a transmission electron microscope (H-7600, Hitachi, Japan).

Size-exclusion chromatography. Two hundred M4B midgut regions from 5th instar nymphs were collected in 0.5 mL of 20 mM Tris buffer (pH 8.0) and homogenized using a microtube homogenizer. Homogenates were centrifuged, and the supernatant was filtered through a 0.45 μm pore filter. The filtered lysate was loaded onto a Superdex 200 (1 cm × 30 cm) column equilibrated with Milli-Q water

and eluted with 30 mL of Milli-Q water at a 0.5 mL/min flow rate. Each fraction contained 1 mL of the eluate. The UV absorbance at 280 nm (OD_{280}) was measured to estimate the protein contents in the eluates. For the symbiont bactericidal assay, *Burkholderia* symbionts isolated from M4 midgut were used. Ten microliter of symbiont solution (OD_{600}, 0.1) was incubated with 10 µL of eluate fraction for 1 h at room temperature. After 1 h incubation, 380 µL of Yeast Glucose (YG) medium (0.5% [w/v] yeast extract, 0.4% [w/v] glucose, and 0.1% [w/v] NaCl) containing 30 µg/mL rifampicin was added to the sample and cultured for 12 h at 30°C. The optical density at 600 nm (OD_{600}) of each sample was compared with OD_{600} of the control, which was prepared using the first eluate fraction. The bactericidal rate was calculated using the following formula: $\{1 - (OD_{600} \text{ of sample} / OD_{600} \text{ of control})\} \times 100$.
Fractions with bactericidal activity from the first size exclusion chromatography (5 mL) were pooled and concentrated to approximately 1/10th the volume using centrifugal concentrators for the second size exclusion chromatography analysis. Prior to loading onto the column, 250 µL of the concentrated sample was treated with an enzyme solution (final concentrations of 100 µg/mL protease K, 100 µg/mL DNase I, or 100 µg/mL protease K and 100 µg/mL DNase I in proper buffer) for 1 h at room temperature. A concentrated sample without enzyme treatment was used as the control. After enzyme treatment, the samples were loaded onto a Superdex 200 column equilibrated with Milli-Q water. Similar to the first size-exclusion chromatography, eluates were obtained by elution with Milli-Q water at a flow rate of 0.5 mL/min and were analyzed for OD_{280} and symbiont bactericidal rates.

Sodium dodecyl sulfate polyacrylamide gel electrophoresis (SDS-PAGE) analysis. To assess the effect of protease K on the M4B fraction, 6 mL of eluate fractions with bactericidal activity from the first chromatography were concentrated to 0.5 mL using centrifugal concentrators. Twenty microliters of concentrated fraction were treated with 100 µg/mL protease K for 2 h at room temperature, and then mixed with reducing Laemmli sample buffer (LSB; 60 mM Tris-HCl [pH 6.8], 2% SDS, 10% glycerol, 0.005% Bromophenol blue) with 2% β-mercaptoethanol. After boiling, the samples were loaded onto a 15% sodium dodecyl sulfate-polyacrylamide gel for electrophoretic analysis. BSA (10 µg) was treated in the same way as the M4B fractions to verify the proteolytic activity of protease K. To completely denature the M4B fraction prior to protease K treatment, reduced LSB was added to the M4B sample and boiled for 1 min. The cooled sample was treated with 100 µg/mL protease K for 2 h and subjected to SDS-PAGE analysis.
For protein and saccharide analyses of the M4B fraction, three fractions with peak OD_{280} values from the second size-exclusion chromatography were chosen for SDS-PAGE. Trichloroacetic acid precipitation was performed to concentrate each M4B fraction. The concentrated samples were prepared under two conditions, denaturing and non-denaturing conditions, before loading onto a 13% polyacrylamide gel. Denaturing conditions used reducing LSB to the samples, followed by boiling for 1 min. Non-denaturing conditions included non-reducing LSB without β-mercaptoethanol and no boiling. Proteins separated on the gel were visualized using Coomassie Brilliant Blue G 250 (Sigma-

Aldrich, USA). Saccharides in the gels were visualized using a Pro-Q Emerald 300 Lipopolysaccharide Gel Stain Kit (Invitrogen, USA).

Analysis of protease inhibitor effects on cathepsin L and bactericidal activity of M4B solution. The protease activity of cathepsin L in the M4B midgut solution was quantitatively analyzed using the synthetic substrate Z(carbobenzyloxy)-Phe-Arg-MCA (methylcoumaryl-7-amide), which is hydrolyzed by cathepsin L and releases fluorescent aminomethylcoumarin. The bactericidal activity of the M4B solution was determined by CFU assay. Firstly, 10 μL of M4B fractions with antibacterial activity collected from size exclusion chromatography was mixed with 10 μL of 40 μM protease inhibitors (E-64, leupeptin, or PMSF) and incubated for 15 min at room temperature. As a positive control, 10 mM phosphate buffer (PB, pH 7.0) was mixed with the M4B fraction instead of the inhibitors. After 15 min of incubation, two assays were performed using this mixture. To determine cathepsin L activity, 400 μL of the Z-Phe-Arg-MCA substrate solution (50 μM substrate in PB) was added to the mixture. After incubation for 10 min at room temperature, 700 μL of 17% (v/v) acetic acid was added to terminate the enzymatic reaction. Cathepsin L activity was detected using a fluorescence spectrophotometer at excitation and emission wavelengths of 350 and 450 nm, respectively. To determine bactericidal activity, 10 μL of bacterial solution containing approximately 500 CFUs of *Burkholderia* symbionts in PB was added to the mixture of antibacterial M4B fractions (10 μL) and protease inhibitor solution (10 μL). For control, the same volume of PB was added instead of protease inhibitor solution. After incubation for 30 min at room temperature, the samples (total 30 μL) were plated on YG agar plates containing 30 μg/mL rifampicin and cultured for 2 days at 30°C to count CFUs. Bactericidal activity (%) of M4B fraction was calculated using the following formula: $\{1 - (\text{CFUs of sample}/\text{CFUs of control})\} \times 100$.

M4B paraffin section staining with Hematoxylin and Eosin (H&E) or Periodic acid-Schiff (PAS). Dissected M4B cells were fixed with 10% formalin for 24 h and dehydrated using an ethanol gradient. After incubation in xylene twice for 1 h each, the tissues were embedded in melted paraffin. The paraffin sections were cut at 4 μm thickness. For staining, paraffin sections were deparaffinized twice in xylene for 6 min at room temperature. Sections were rehydrated in 100, 95, and 70% ethanol for 2 min each, followed by washing in distilled water. A H&E staining kit (VitroVivo Biotech, USA) was used for H&E staining. Sections were incubated in Mayer's hematoxylin solution for 5 min and rinsed thrice with tap water. Subsequently, the sections were placed in 95% ethanol for 1 min and then in eosin for 1 min 30 sec.

Periodic acid-Schiff (PAS) staining was performed using a PAS staining kit (VitroVivo Biotech, USA). After oxidation with 1% periodic acid for 5 min, sections were briefly washed with distilled water. The sections were incubated in Schiff's reagent for 15 min and washed with lukewarm tap water for 5 min. Counterstaining was performed with Mayer's hematoxylin for 1 min and the sections were then washed with distilled water. After staining, the sections were dehydrated in an ethanol gradient and cleared with

xylene. Finally, sections were mounted and sealed with glass coverslips.

M4B smear staining with Periodic acid-Schiff (PAS). M4B midgut dissected from 5th nymphal instar bean bug was smeared on a glass slide by pressing with a pipette tip. After air-drying at room temperature for 10 min, the smear was slightly heat-fixed. Then, the smear was incubated with 1% periodic acid for 5 min and washed with distilled water. The smear was incubated in Schiff's reagent for 1 min and washed with lukewarm tap water for 5 min. Finally, the smears were dehydrated and cleared with xylene. Stained smears were mounted and sealed with glass coverslips.

Ultra high resolution low voltage field emission scanning electron microscopy. One hundred microliter of *Burkholderia* symbionts isolated from M4 regions of 5th instar nymphs (OD_{600}, 1.0) was incubated with 100 μL of eluted M4B fraction for 2 h at room temperature. After incubation, the samples were pre-fixed with 2.5% paraformaldehyde and 2.5% glutaraldehyde in phosphate buffer with saline (PBS, pH 7.2) at room temperature for 2 h. After pre-fixation, samples were washed three times with PBS and post-fixed with 1% osmium tetroxide in PBS at 4°C for 1 h. After three times of washing with PBS, the samples were dehydrated with graded ethanol series and isoamyl acetate. The sample was placed on a microscope glass slide and then air dried. Ultra high resolution low voltage field emission scanning electron microscopy (HRLV-SEM, JEOL, JSM-7900F, Japan) (Metabolomics Research Center for Functional Materials, Kyungsung University, South Korea) was used to examine the surface morphology of the prepared sample.

Figure S1. Effect of protease inhibitors on cathepsin L activity and bactericidal activity of M4B eluates. (A) Cathepsin L activity of M4B eluates detected by fluorescent substrates was inhibited by cathepsin L inhibitors. Cathepsin L specific inhibitors, E-64 (N-[N-(L,trans-carboxyoxiran-2-carbonyl)-L-leucyl]agmatine) and leupeptin, abolished cathepsin L activity of M4B eluates. However, the typical serine protease inhibitor PMSF (phenylmethylsulfonyl fluoride) did not reduce cathepsin L activity. **(B)** Inhibition of cathepsin L activity has no effect to the bactericidal activity of M4B eluates. M4B eluates with abolished cathepsin L activity by cathepsin L inhibitors, E-64 and leupeptin, exhibited unaltered bactericidal activity.

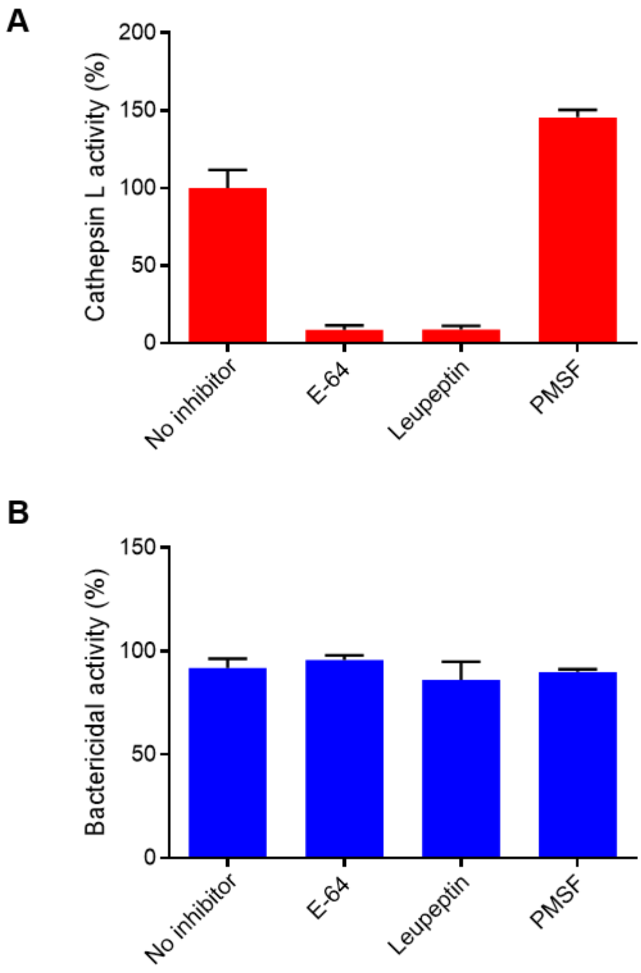

Figure S2. Transmission electron microscopy images of symbiotic organs. The lumen of M4 midgut region of symbiotic bean bug (**A**) is filled with *Burkholderia* symbionts. Both M4B region of symbiotic bean bug (**B**) and M4B region of aposymbiotic bean bugs (**C**) have longer villi than M4 region. However, the lumen of symbiotic M4B is denser than that of aposymbiotic M4B.

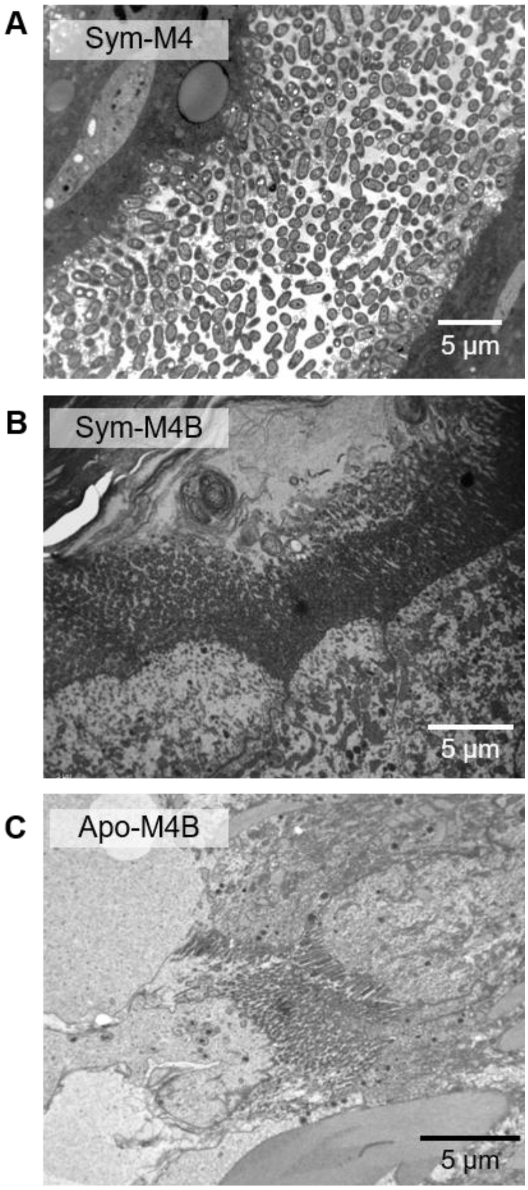

Figure S3. (A) Size-exclusion chromatography of M4B fractions treated with DNase I and/or protease K treatment. Prior to loading onto a Superdex 200 column, eluates with bactericidal activity from the first size-exclusion chromatography were treated with DNase I **(i)** or DNase I and protease K for 1 h **(ii)**. The protein contents of eluates were detected by UV absorbance at 280 nm (OD_{280}, red line). Bactericidal activity of each fraction was determined by optical density at 600 nm of a 12 h culture of *Burkholderia* symbiont after treating with fractions (light blue column). **(B) Non-denaturing SDS-PAGE analyses of M4B fractions with protein or DNA staining.** Proteins and DNAs on SDS-PAGE gels were visualized by Coomassie Brilliant Blue staining and ethidium bromide staining, respectively. The HMW complexes are shown in a non-denaturing gel in protein staining, but no bands were observed in DNA staining.

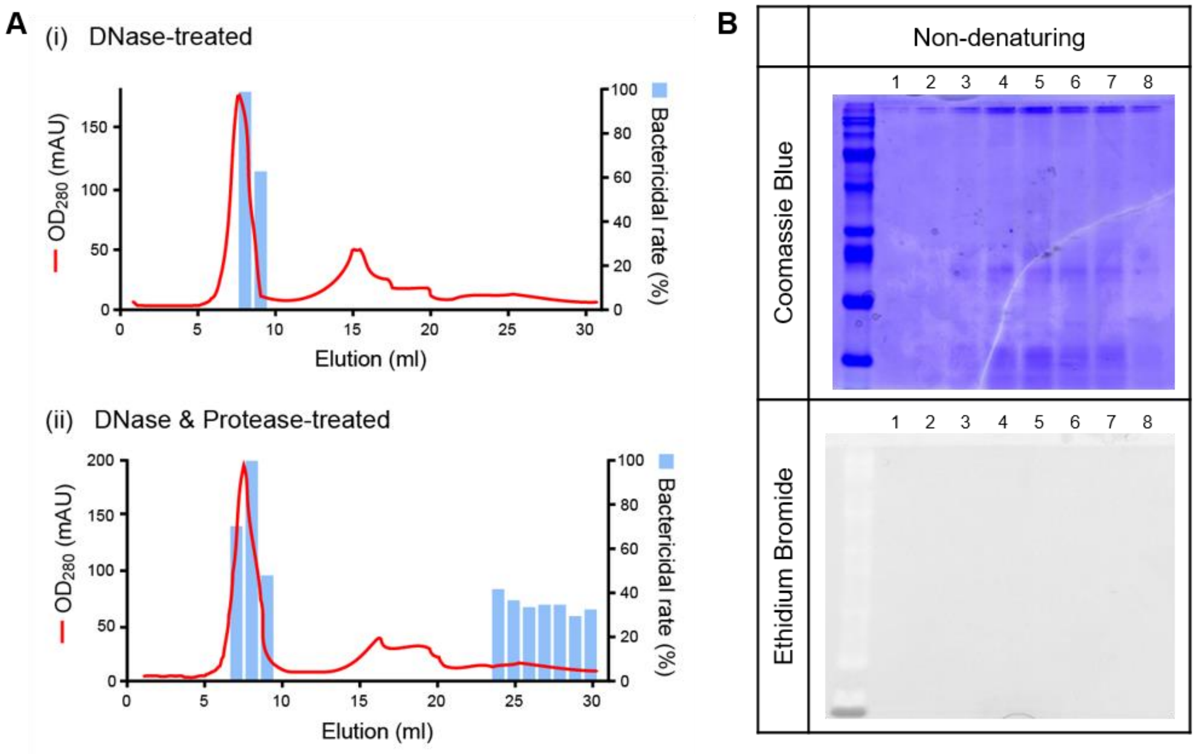

Figure S4. Scanning electron microscopy (SEM) images of *Burkholderia* symbiont cells. *Burkholderia* symbiont cells were untreated (**A**) and treated with M4B eluates (**B**). *Burkholderia* symbiont cells were lysed with treatment of M4B.

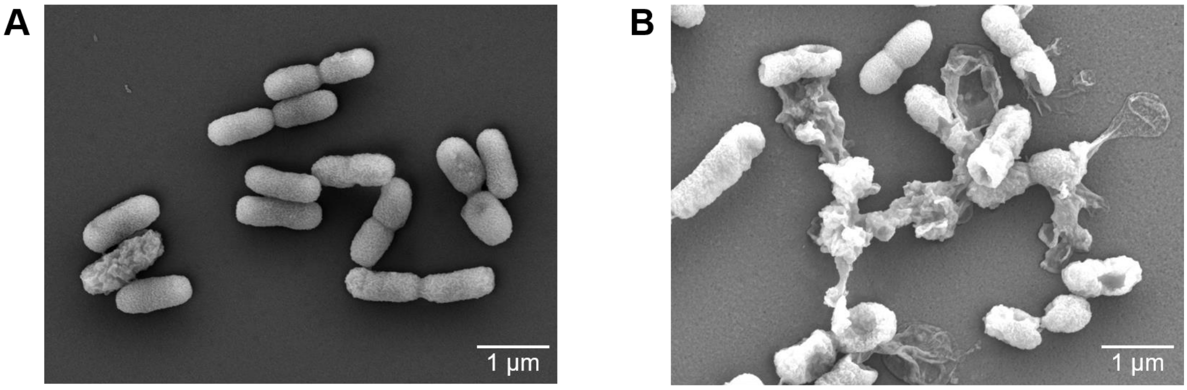

Supplement: M4B_supplementary_information_wrad021 [file m4b_supplementary_information_wrad021.pdf]
